# Three-Axis Vector Magnetometer with a Three-Dimensional Flux Concentrator

**DOI:** 10.3390/s24051659

**Published:** 2024-03-04

**Authors:** Shih-Jui Chen, Der-Tai Hong, Ping-Hsun Hsieh, Tse-Kang Wang

**Affiliations:** Department of Mechanical Engineering, National Central University, Taoyuan 320317, Taiwan

**Keywords:** flux concentrator, magnetometer, three-axis

## Abstract

This research proposes a magnetic field sensor with spatial orientation ability. Through the assistance of a magnetic flux concentrator, out-of-plane magnetic flux can be concentrated and guided into the planar magnetic cores of a fluxgate sensor. A printed circuit board is used to construct the basic planar structure, on which the proposed three-dimensional magnetic flux concentrator and magnetic cores are assembled. This reduces the alignment error of the coils and improves the reliability of the sensor. Three-axis sensing is achieved by using the second harmonic signals from selected sensing coil pairs. The magnetometer exhibits a linear range to 130 μT. At an excitation frequency of 50 kHz, the measured sensitivities are 257.1, 468.8, and 258.8 V/T for the X-, Y-, and Z-axis sensing modes, respectively. This sensor utilizes only one sensing mechanism for the vector field, making it suitable for IoT applications, especially for assessing mechanical posture or position.

## 1. Introduction

With the rapid development of technology, intelligent IOT sensors that integrate the production process with virtual and real sceneries are widely studied. Smart sensing generally includes parameter measurement, data transmission, and data calculation. Take a magnetometer as an example. Common magnetic field sensing methods include the Hall effect, magnetoresistive method, and fluxgate method [1,2]. Hall effect sensors are often used to measure the earth’s magnetic field and motor position [3,4]. Magnetoresistive and fluxgate sensors are often used in satellite navigation and medical treatment [5,6,7]. The factors involved in developing a vectorial magnetometer are miniaturization, low power consumption, and cost considerations. To improve the performance of the sensor, various material technologies and manufacturing methods have been developed, including printed circuit board (PCB) and microelectronics technology [8,9,10].

The purpose of this study is to develop a tri-axis magnetometer that can measure a vector magnetic field in space. Several studies have been presented, using a combination of either same or different types of sensors [11,12]. There is also an element that uses a single permalloy core and two excitation coils to achieve three-axis sensing [13]. To measure the magnetic field perpendicular to the sensor, Silva [14] deposited a layer of magnetoresistive material on a micromachined v-groove surface for z-axis sensing. Hsieh [15] applied an inverted V-shaped flux conductor on a silicon substrate to assist the out-of-plane sensing. These sensors can be further integrated with in-plane sensors.

An integrated tri-axial sensor can either reduce the complexity of the component placement or working circuit. To increase magnetic flux in a specific direction, flux guide concentrators are introduced into magnetic sensors [15,16,17,18]. Made of soft ferromagnetic materials with high permeability, these concentrators can effectively conduct the out-of-plane magnetic flux lines to the element plane for z-axis direction sensing. Zhao [19] designed a slope magnetic flux guide on a silicon substrate for out-of-plane magnetic sensing with a planar GMR sensor. Lu [20] proposed a tri-axial sensor with a flux guide tube for collecting an orthogonal magnetic flux. Considering manufacturing compatibility, this paper uses a three-dimensional flux concentrator to conduct a z-axis magnetic field to a planar fluxgate. It eliminates the requirement for multiple driving circuits commonly used in multi-sensor configuration, thereby reducing power consumption. The material of the flux concentrator and magnetic core is an amorphous Co-based alloy, VITROVAC 6025 Z. Measurement results show that the sensitivity in the z-axis is increased by applying a magnetic concentrator, and three-axis sensing is achieved with the proposed combination of coils and magnetic cores.

## 2. Design

The sensor’s performance mainly depends on the magnetization state of the magnetic core and the conduction efficiency of the flux concentrator. To increase the induced electromotive force (EMF) in the coil, a nearby magnetic core can be used to conduct a suitable magnetic field. Typically, the magnetic flux density of a coil can be expressed by $B_{coil}=\mu H_{ex}$*,* where μ is the permeability and $H_{ex}$ is the magnetic field strength produced by an excitation coil. The excitation magnetic field generated by the excitation coil will affect the magnetization in the core. Moreover, there is angle difference between the direction of the magnetic flux passing through the induction coil and the area vector of the coil. Therefore, the aforementioned equation can be rewritten as

(1) $$B_{coil}=\eta \mu H_{ex},$$

where η is the effective ratio.

**Figure 1:**
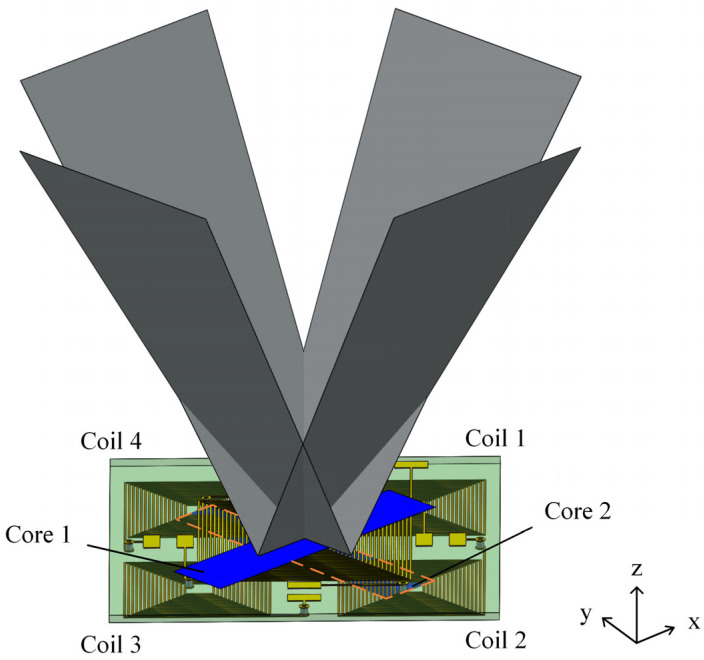
Schematic of the proposed three-axis magnetometer.

In this research, we propose a magnetometer with three-axis magnetic field sensing ability. The basic planar structure is composed of an excitation coil, four induction coils, a set of magnetic cores, and a three-dimensional magnetic flux concentrator, as shown in Figure 1.

**Figure 2:**
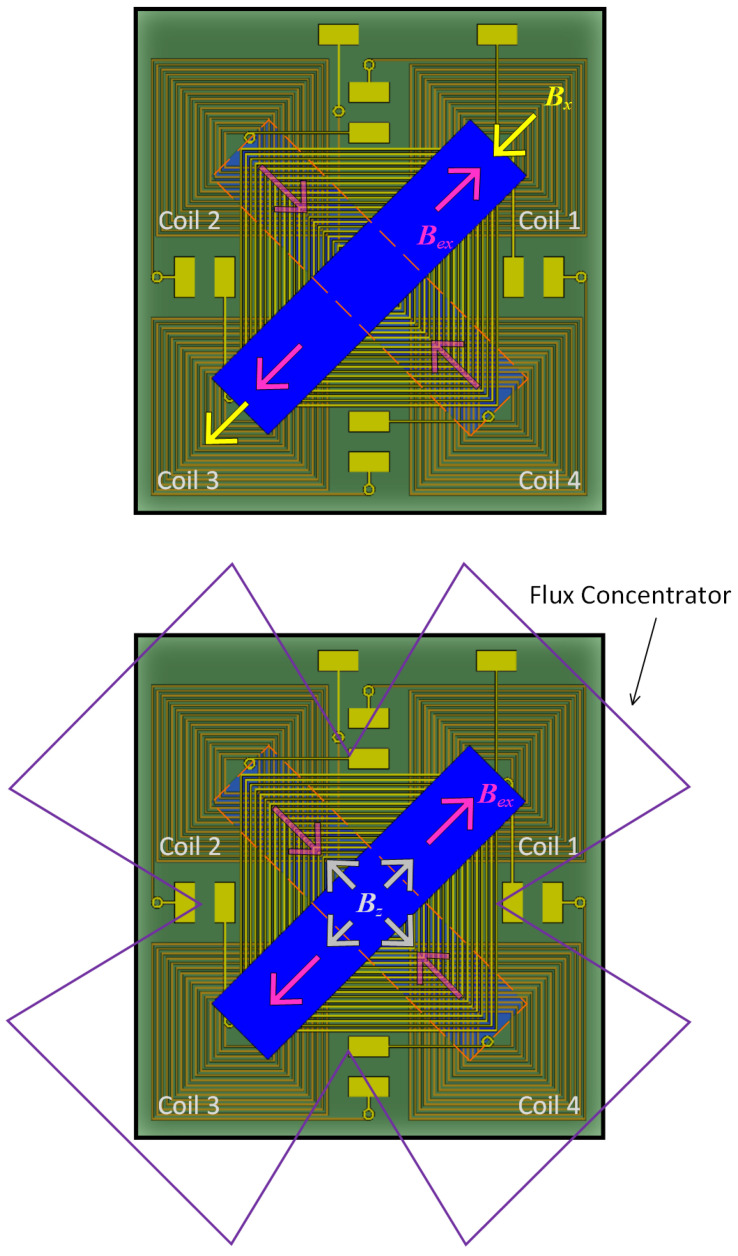
Schematic diagram of the magnetic field directions in the core under different external magnetic fields: (**upper**) ***B***x; (**lower**) ***B***z.

Figure 2 illustrates how the magnetic field within a magnetic core aligns under the influence of various external magnetic fields. The red arrow in the diagram represents the magnetic field that is generated by the excitation coil, while the yellow arrow indicates the external magnetic field. If a magnetic field is applied along the x-axis of the core, the magnetic field lines within the core will become parallel to the x-axis. The external magnetic field and the magnetic field produced by the induction coil on the right side of the core are in opposite directions. However, on the left side, the core’s magnetic field aligns with the external magnetic field. Therefore, coil pair 1–3 can be chosen as an in-plane sensing coil pair. When the external magnetic field is in the z direction, it is guided downward along the magnetic flux concentrator. The external magnetic field at coil 1 is in the same direction as the excitation field, while the external magnetic field at coil 2 is in the opposite direction to the excitation field. Therefore, coil pair 1–2 can be selected as the sensing coil pair.

**Figure 3:**
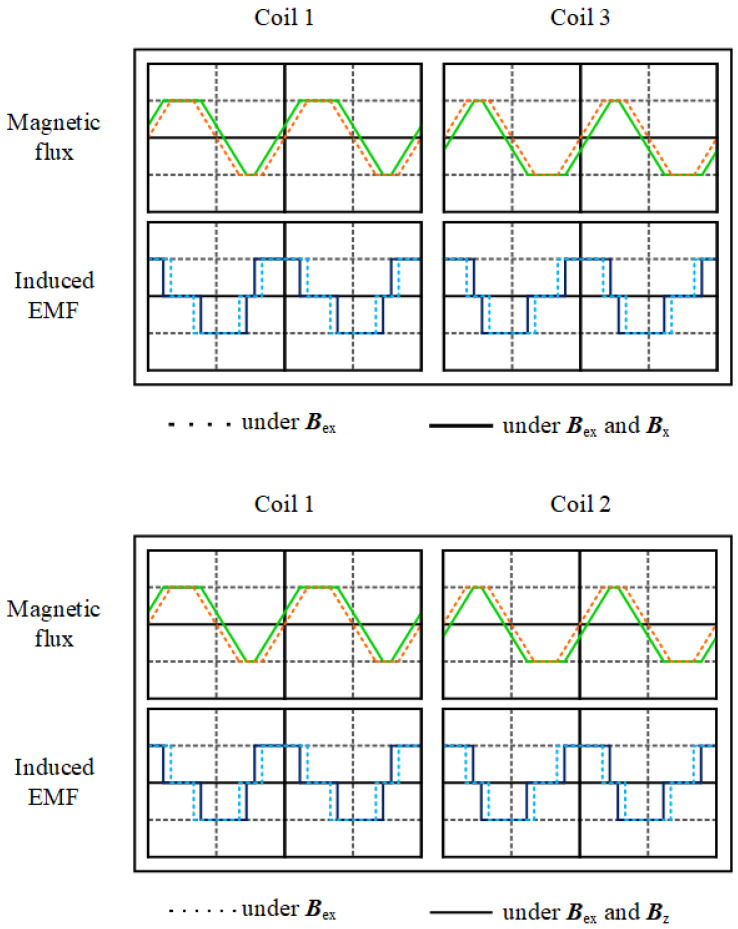
The magnetic flux and induced EMF of the coils under the influence of ***B***x (**upper** figure) and ***B***z (**lower** figure).

When the external magnetic field is in the x direction, both magnetic fields at coil 1 align, resulting in an increase in magnetic flux. Conversely, the magnetic fluxes at coil 3 decrease. By using Faraday’s law, the induced emf can be obtained from the time derivative of the magnetic flux. As shown in Figure 3, the induced EMF curves of coils 1 and 3 shift in opposite directions. Subtracting the two curves results in the generation of a second harmonic waveform.

When the external magnetic field is in the z direction, the induced EMF curves of coils 1 and 2 change in opposite directions. Meanwhile, coils 1 and 3 undergo identical changes in induced EMF as both fields align in the same direction, resulting in a zero output. This prevents interference from the in-plane magnetic field.

The induction coils are combined into pairs for x-, y-, and z-direction sensing, as listed in Table 1. For example, when subjected to an x-direction magnetic field, coils 1 and 3 are selected as a sensing coil pair, and a second harmonic waveform can be generated by subtracting the waveform of coil 3 from the waveform of coil 1. Similarly, when subjected to a z-direction magnetic field, coils 1 and 2 are selected as a sensing coil pair. Therefore, selecting different coil pairs for sensing enables three-axis magnetic field sensing.

## 3. Simulation

**Figure 4:**
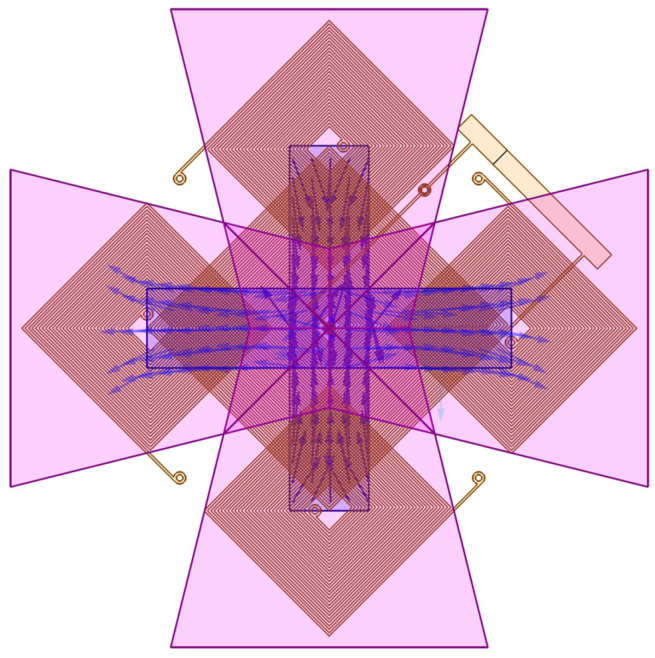
The direction of the excitation magnetic field in the cores.

The magnetization of cores will be affected by the external magnetic field and the excitation magnetic field. As a result, the induced electromotive force of the corresponding induction coils will change. This research uses Ansys Maxwell software 2020 for magnetic field simulation. According to the specifications of the magnetic core, the saturation magnetic flux density is set to 0.58 T. Figure 4 illustrates the simulation of the magnetic field generated by the excitation coil. Since the excitation coil is sandwiched between the horizonal and vertical cores, the magnetization directions of the cores are opposite. In this way, the excitation magnetic field required for three-axis sensing is created. Even if a magnetic conductor is added, the proposed excitation coils can still effectively build opposite magnetization at ends of the cores.

**Figure 5:**
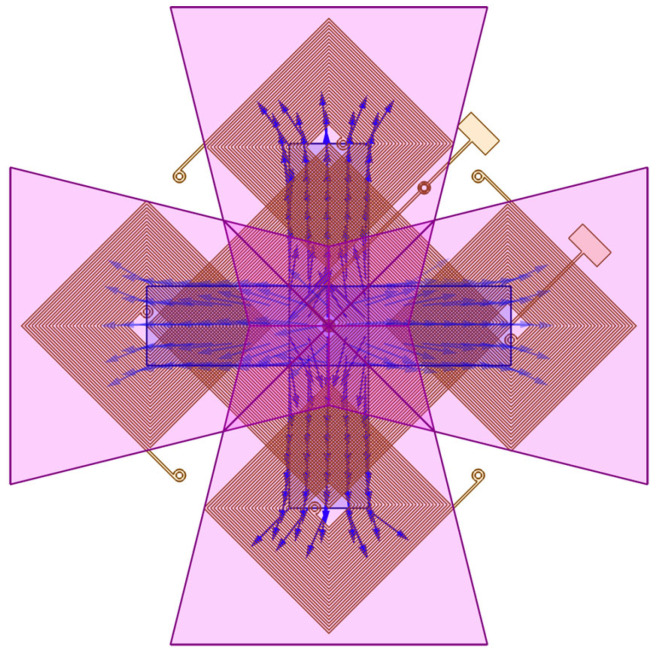
The direction of the magnetic field in the cores in response to the z-axis magnetic field.

Next, simulations are performed to show whether the magnetic conductor can conduct the out-of-plane magnetic flux to the element plane. Theoretically, the conduction efficiency of the flux concentrator will determine the z-axis sensing capability of the proposed sensor. As shown in Figure 5, the z-axis magnetic field is guided to the ends of the cores, and the magnetization directions of both cores are the same.

**Figure 6:**
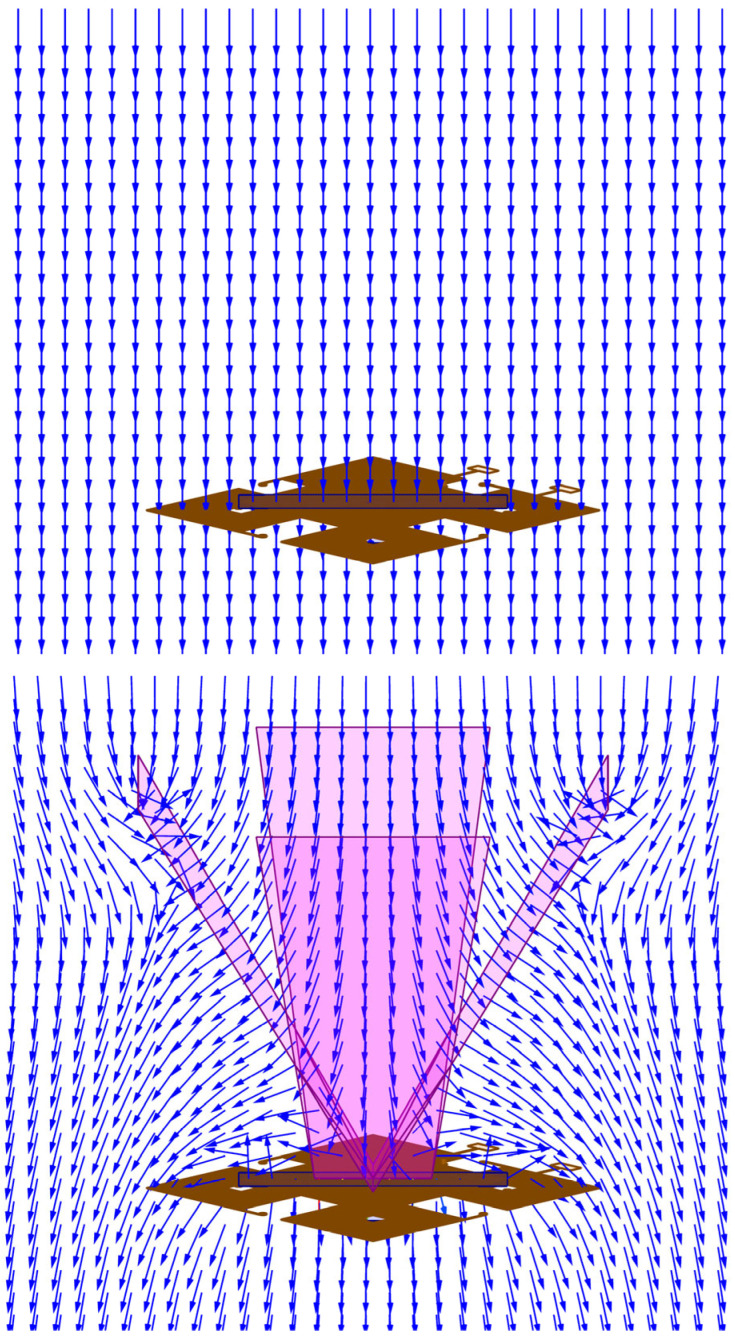
The distribution of the out-of-plane magnetic field applied to a magnetometer without a flux concentrator (**upper** figure) and with a flux concentrator (**lower** figure).

We can compare the magnetic field distribution changes with and without a flux concentrator, as illustrated in Figure 6. Without a flux concentrator, most of the z-axis magnetic field directly penetrates the core, and as a result, the magnetization of the magnetic core remains unaffected. However, when the proposed magnetic concentrator is added, it alters the magnetic field distribution. The flux concentrator can effectively be used to collect out-of-plane magnetic flux and redirect it towards planar cores located at the ends of the flux concentrator. This results in a symmetrical magnetization distribution along the center of the core.

## 4. Sensor Manufacturing

**Figure 7:**
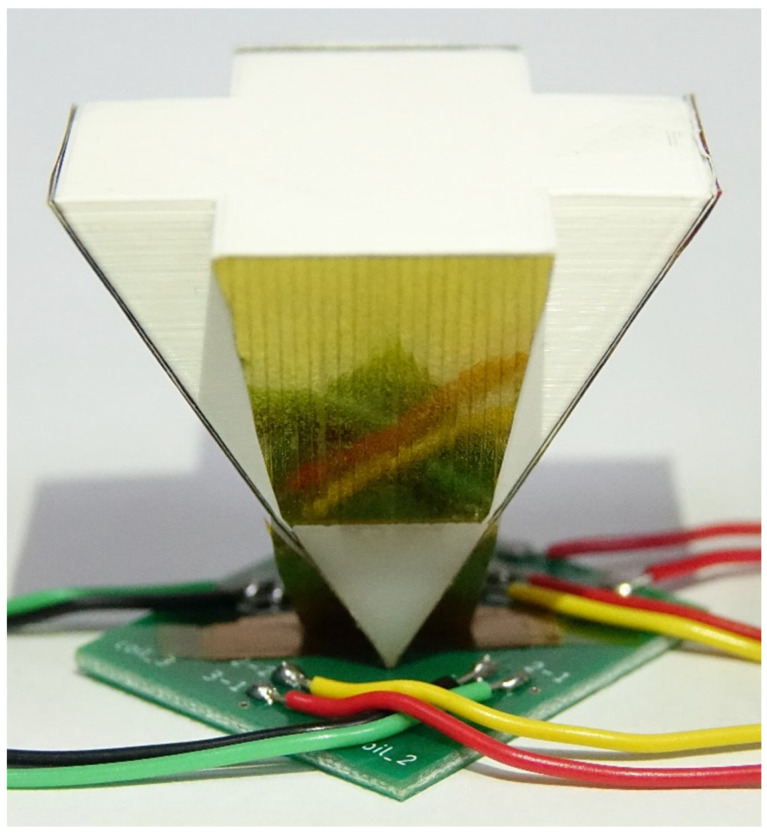
Photo of the PCB-based magnetometer.

The fabricated magnetometer is shown in Figure 7, including the coils, cores, and flux concentrator. The excitation and induction coils are fabricated on a double-sided printed circuit board (PCB) to increase the stability and quality of the element. Subsequently, 3D printing is used to build the basic support for the flux concentrator. The parameters of the sensor are listed in Table 2. Considering the component area and power loss, 25-turn excitation coils and 15-turn induction coils are employed as the basic layout blocks. In the design of the flux concentrator, the angle between the hypotenuse and the element plane is 60 degrees, and the V-shaped structure is designed to be wider at the top and narrower at the bottom to improve the transmission effect. The magnetic flux concentrator and magnetic cores are further assembled on the PCB, which simplifies the process steps. Moreover, a lock-in amplifier circuit is also built on a PCB.

## 5. Testing Setup

**Figure 8:**
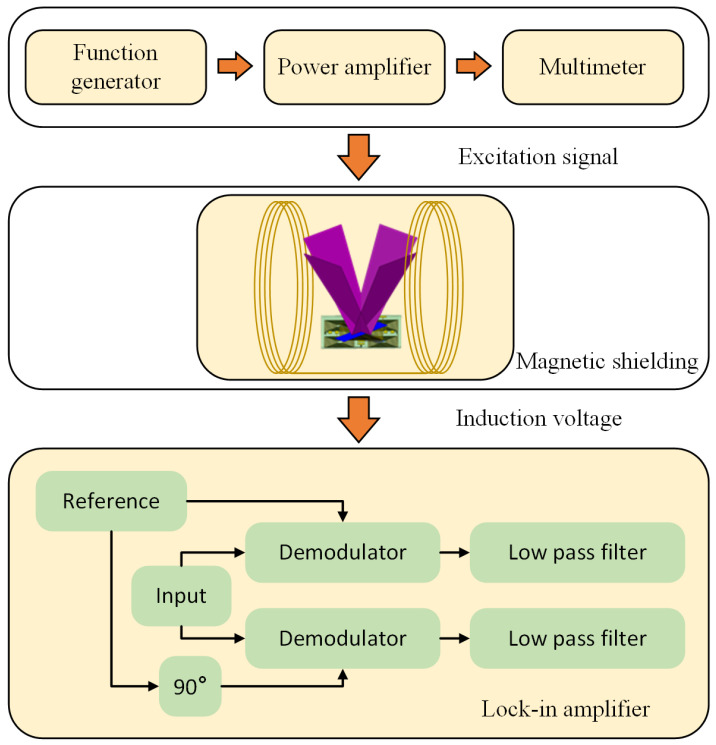
The measurement architecture.

In Figure 8, the three-axis vector magnetometer is shown placed inside a Helmholtz coil for calibration. The magnetometer’s sensing axis was aligned with the magnetic field produced by the Helmholtz coil. An excitation signal was given to the magnetometer by a signal generator and then fine-tuned to a specific current through the use of a power amplifier. The resulting waveforms from each coil when exposed to external magnetic fields were recorded. The subtraction of paired coil signals produced a second-harmonic frequency signal.

A lock-in amplifier was used to extract the required DC signal. To address the issue of phase difference between the input and reference signals, the reference signal was split into two signals with a 90-degree phase offset. The modulator multiplied the signals, and the resulting signal was then passed through a low-pass filter to generate a DC voltage output. Throughout the measurement, the Helmholtz coil was placed in a magnetic shield to eliminate external stray magnetic fields.

## 6. Measurement Results

For the magnetic field sensing, the parameters of the magnetic core and the exciting current obviously affect the sensitivity and linear range. Based on the Ref. [12], a magnetic core of 5 mm was used for the following measurements. Due to the limitation of the IC used in the lock-in amplifier circuit, 50 kHz was set as the upper limit of the frequency range. The exciting current was set to 700 mA to prevent overheating the element.

**Figure 9:**
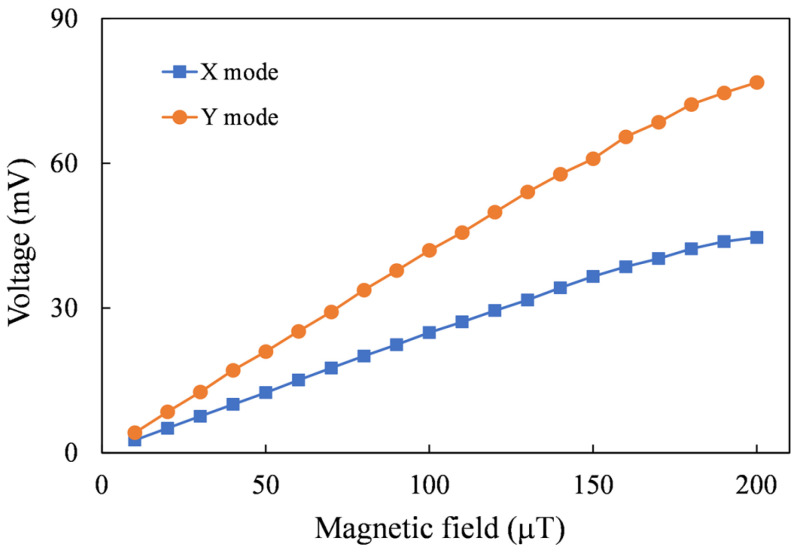
Measured voltages induced by two axial magnetic fields.

To ensure the effective magnetization of the magnetic core by the excitation magnetic field, initial testing was conducted without an assisted flux concentrator. It is observed from Figure 9 that the sensitivity of X-axis mode is 241.1 V/T and the sensitivity of Y-axis mode is 409.8 V/T.

### 6.1. Three-Axial Magnetic Field Sensing

**Figure 10:**
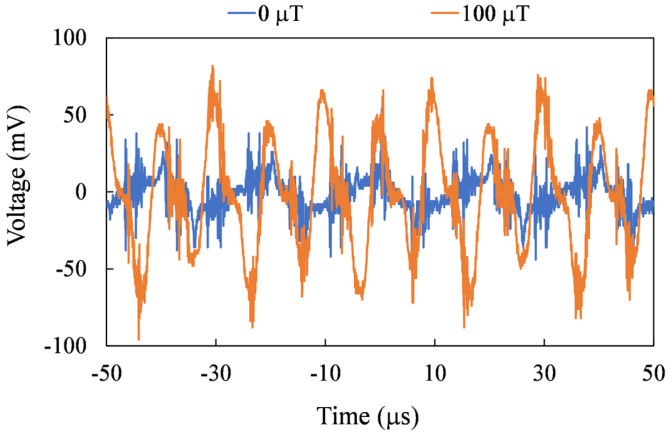
Measured second harmonic waveforms for the X-axis mode.

**Figure 11:**
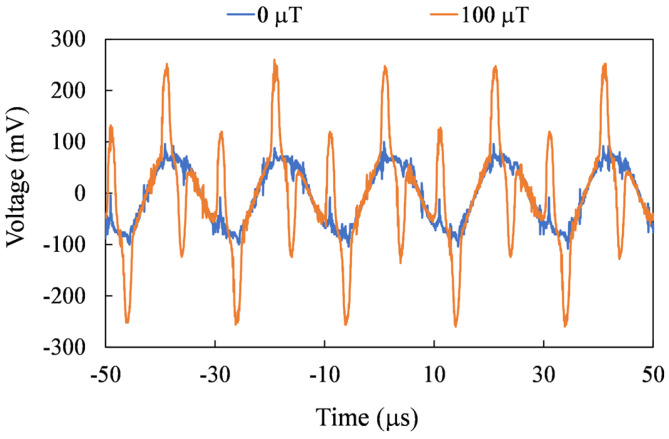
Measured second harmonic waveforms for the Y-axis mode.

**Figure 12:**
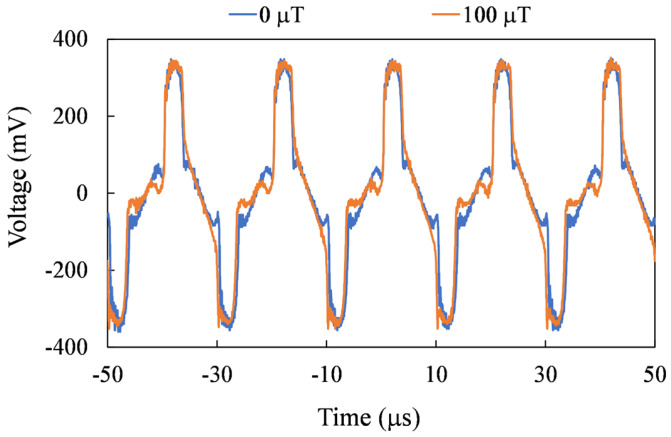
Measured second harmonic waveforms for the Z-axis mode.

For precise spatial magnetic field measurements, the sensor needs to be able to measure on three axes. In Figure 10, Figure 11 and Figure 12, the second harmonic waveforms of each in pair are depicted under the influence of three axial external magnetic fields. It is evident that the second-harmonic signal becomes more pronounced with the increasing strength of the external magnetic field. Furthermore, even in the presence of an external magnetic field, the second harmonic signal is not noticeable for the non-corresponding sensing axes.

**Figure 13:**
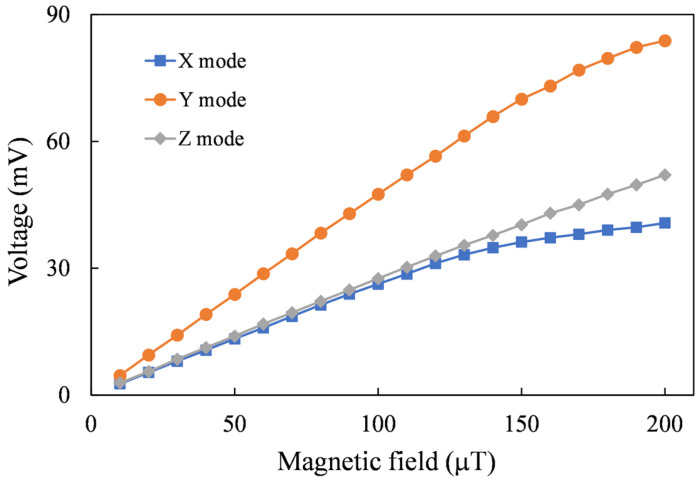
Measured voltages induced by three axial magnetic fields.

The measured waveforms were processed by a lock-in amplifier circuit. The converted dc voltages with respect to axial magnetic fields ranging from 0 to 200 μT are plotted in Figure 13. For the output voltage, the region where the nonlinearity is less than 10% is defined as the linear range, as listed in Table 3. The average voltage to magnetic field conversion ratio in this range is the sensitivity. The measured sensitivities are 257.1 V/T for the X-axis sensing mode, 468.8 V/T for the Y-axis sensing mode, and 258.8 V/T for the Z-axis sensing mode. It is found that the sensitivity of the y-axis is higher than that of the x-axis. The main reason is that the y-axis core is closer to the induction coils, while the x-axis core is about 1.6 mm away from the induction coils. Moreover, it can be observed that beyond the linear range, the sensitivity gradually decreases with the increasing magnetic field.

For a three-axis sensor, a magnetic field applied to a single axis will be sensed by other axes, which is called coupling. To analyze the coupling between the axes, a single-axis magnetic field was applied and the output of three sensing modes were recorded. In Z mode, the x-axis and y-axis magnetic field couplings are 1.22% and 1.79%, respectively.

### 6.2. Directional Magnetic Field Sensing

**Figure 14:**
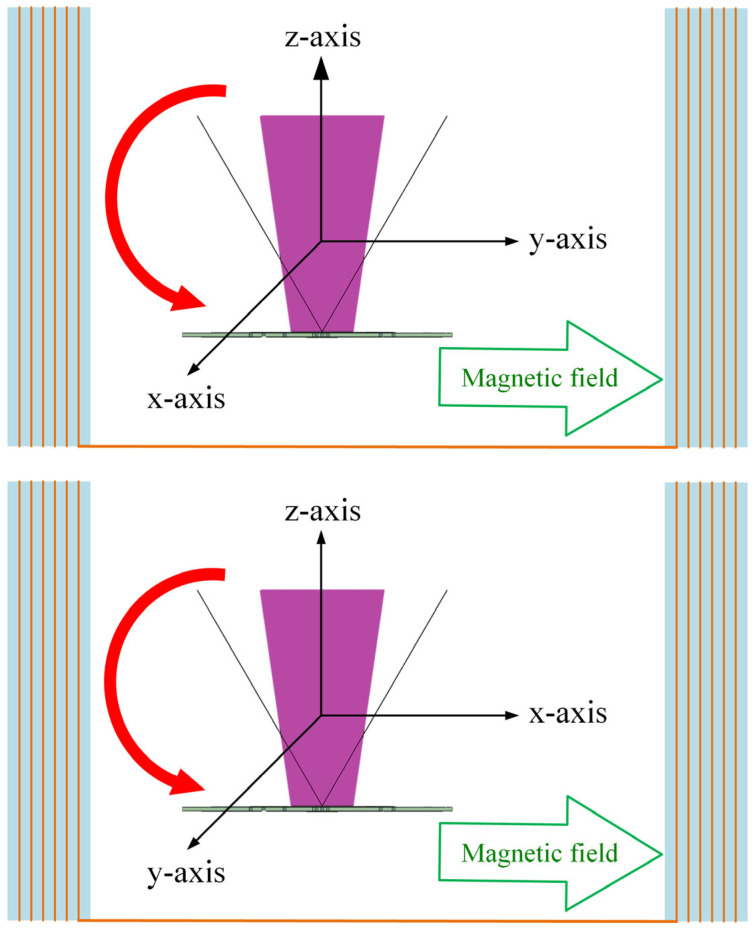

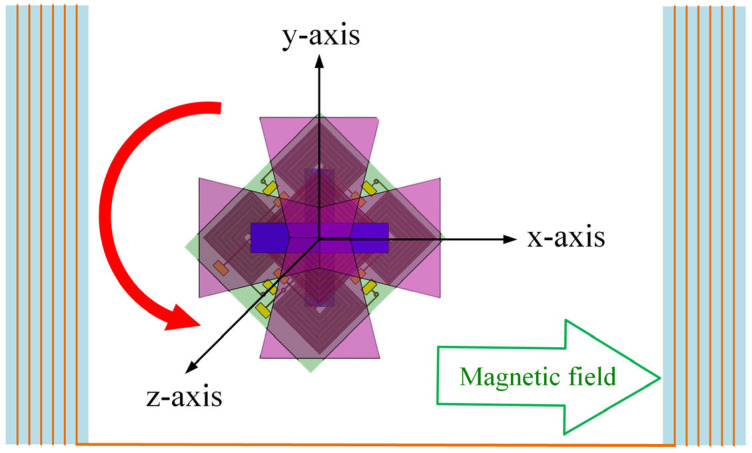
Illustrations of sensor angle measurements in magnetic fields in the y–z (**top** figure), x–z (**middle** figure), and x–y (**bottom** figure) planes.

**Figure 15:**
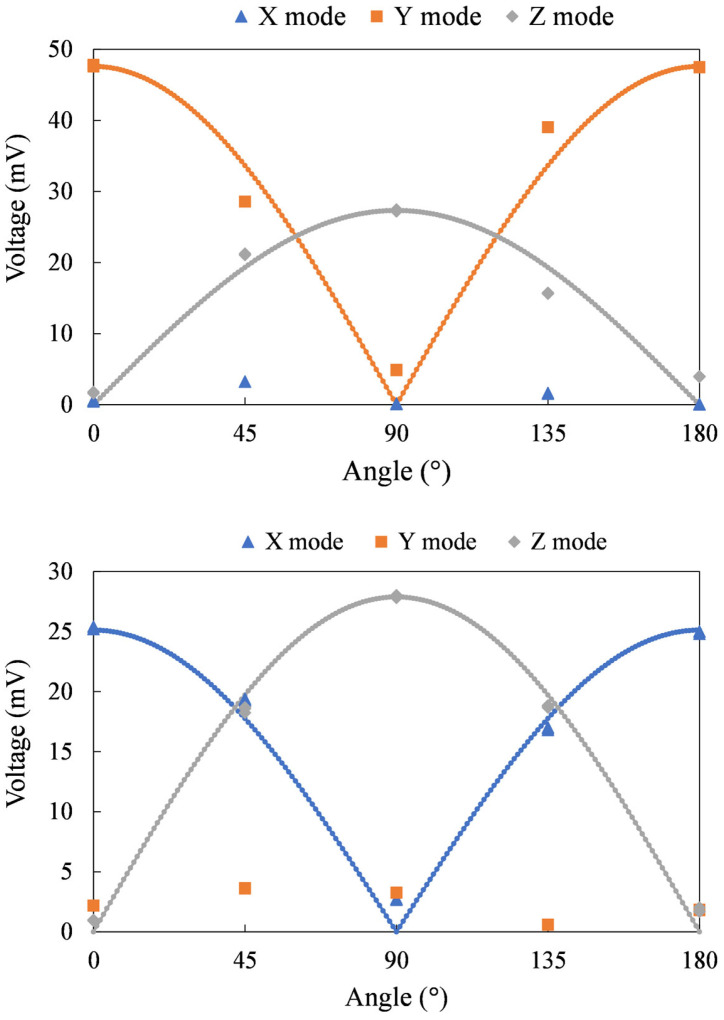

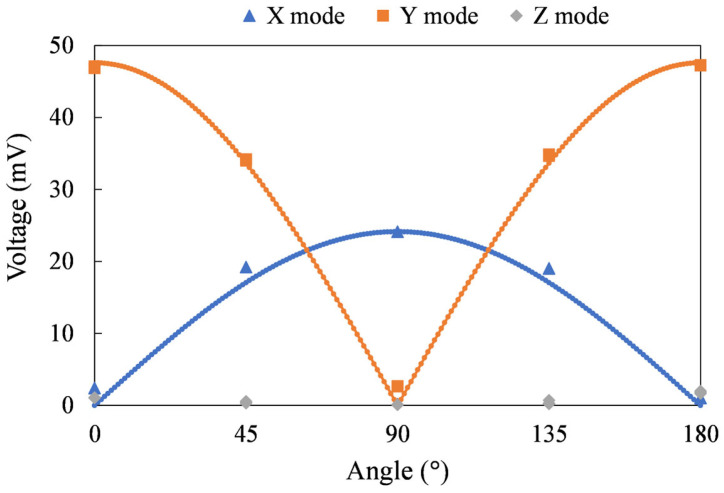
Output voltage for the sensor in a magnetic field in the (**top**) y–z, (**middle**) x–z, and (**bottom**) x–y planes. The blue, orange, and grey lines refer to the predicted values of the X mode, Y mode, and Z mode, respectively.

For three-axis sensors, vector magnetic field sensing capabilities are required. During the measurement, the sensor was rotated along the x, y, and z axes of the sensor, as shown in Figure 14. The voltage measured at an external magnetic field of 100 µT is plotted against the rotation angle, as shown in Figure 15. The voltage waveforms in the magnetic fields of the y–z plane, x–z plane, and x–y plane exhibit a complete sine wave trend, differing by 90 degrees. The measurement results are consistent with the projection of the magnetic field on the two axes. This confirms the sensor’s ability to sense direction. Angular measurement errors may result from misalignment of the core or concentrator and incorrect sensor positioning within the Helmholtz coil. In forthcoming studies, accuracy can be enhanced by incorporating mechanical fixtures during sensor assembly or calibration processes.

The normalized root mean square error (NRMSE) is used to calculate the similarity between the predicted values (${\hat{y}}_{i}$) and the corresponding measured values ($y_{i}$), as indicated in Equation (2). The decrease in NRMSE reflects the increasing similarity between the two curves. In y–z plane measurements, the NRMSE is 0.108 for Y mode and 0.154 for Z mode. In x–z plane measurements, the NRMSE is 0.075 for X mode and 0.065 for Z mode. In x–y plane measurements, the NRMSE is 0.115 for X mode and 0.039 for Y mode. Measurement errors can be caused by undesired coupling, core and flux concentrator misalignment, and sensor placement errors. Deviations are acceptable.

(2) $$NRMSE=\sqrt{\frac{\sum_{i=1}^{N}{\left(y_{i}-{\hat{y}}_{i}\right)}^{2}}{\sum_{i=1}^{N}{{\hat{y}}_{i}}^{2}}},$$

### 6.3. Noise Analysis

**Figure 16:**
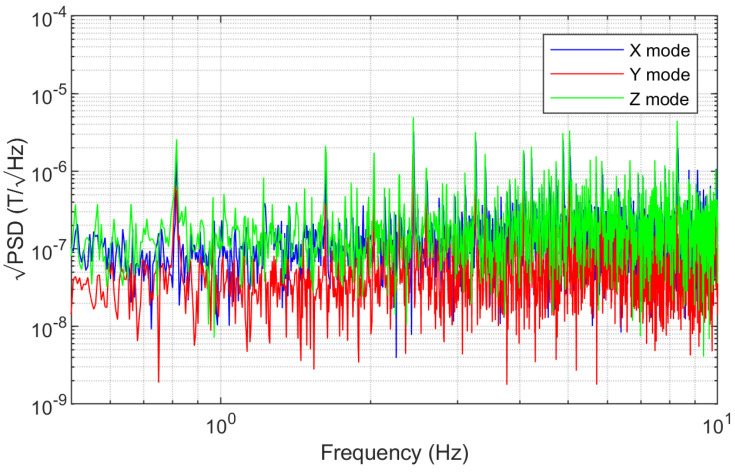
The square root of the sensor’s PSD.

When subjected to an excitation magnetic field in the absence of external magnetic field, the power spectral density of the sensor’s output voltage was analyzed. Figure 16 shows the square root of the noise power spectral density (PSD) in the frequency range of 0.5–10 Hz. The mean $\sqrt{PSD}$ of the X sensing mode is 213 $nT/\sqrt{Hz}$. The mean $\sqrt{PSD}$ of the Y sensing mode is 71 $nT/\sqrt{Hz}$. The mean $\sqrt{PSD}$ of the Z sensing mode is 249 $nT/\sqrt{Hz}$. The noise levels of the X, Y, and Z modes are 1.04 μT, 0.37 μT, and 1.24 μT, respectively. It can be observed that the noise level in the Y mode is the smallest, and this trend is consistent with the higher sensitivity.

### 6.4. Demonstration

**Figure 17:**
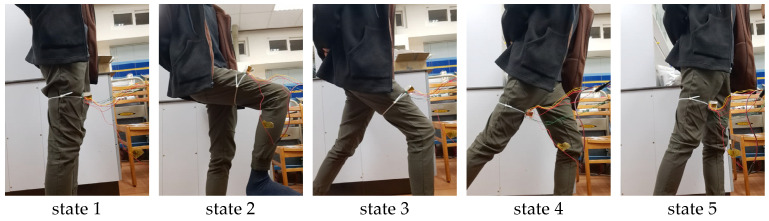
Photographs of simulated walking poses captured from state 1 to state 5.

**Figure 18:**
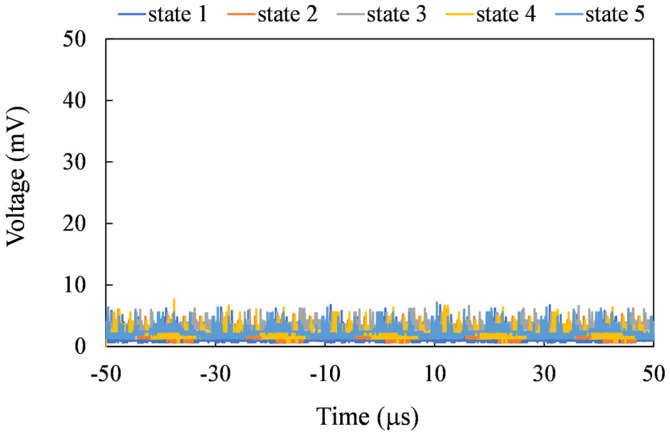

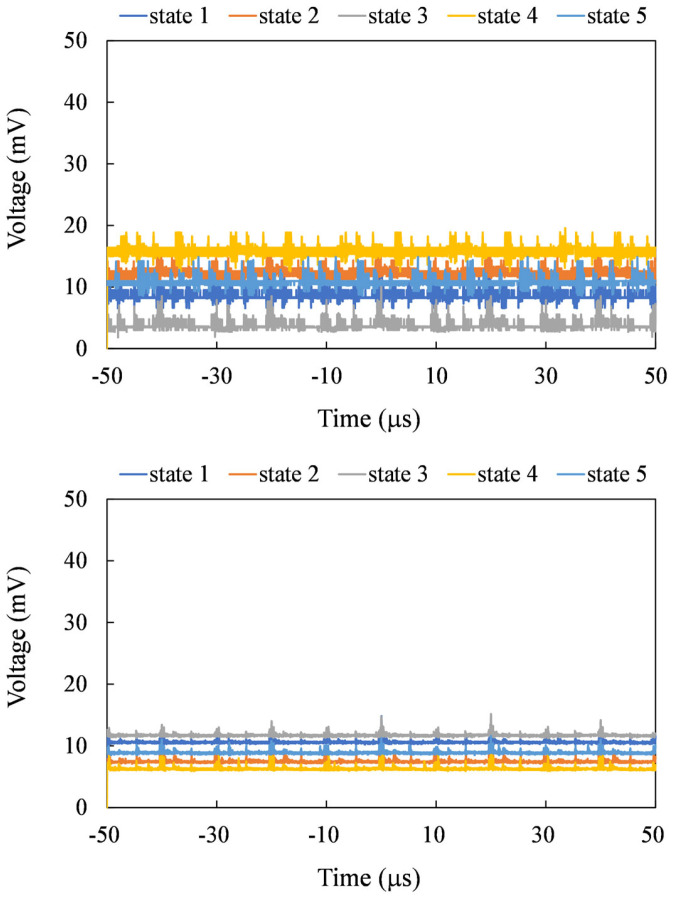
The top, middle, and bottom figures show the output voltages for states 1 to 5 in the X, Y, and Z sensing modes, respectively.

The sensor can be utilized to measure angular variations, exemplified in the assessment of walking posture (see Figure 17). During the demonstration, we roughly aligned the sensor’s y–z plane with the Earth’s magnetic field. In the initial position, or state 1, of the sensor, the z-axis was aligned perpendicular to the thigh, and the y-axis was aligned parallel to the thigh. In Figure 18, the output signals corresponding to states 1 through 5 during walking are presented. It was observed that X-mode sensing produced the least signal. In Z-mode sensing, the signal was highest in state 3 due to the better alignment between the sensor’s z-axis and the Earth’s magnetic field. Similarly, in Y-mode sensing, the highest output is observed in state 4 for the same reason.

## 7. Conclusions

A magnetometer consisting of a planar fluxgate and a flux concentrator is proposed to achieve three-axis sensing. The three-axis sensor only adopts one sensing mechanism, which simplifies the circuit design. With the proposed flux concentrator, the out-of-plane magnetic flux can be effectively collected and transmitted to the planar core as an aid for z-axis sensing. Both in-plane and out-of-plane magnetic fields can be sensed by using different sensing coil pairs. The sensor can be used to measure the angular variations of Earth’s magnetic field, particularly in the assessment of the mechanical posture or position. The experiment results indicate that there may be an optimal condition for the magnetization of the cores and the concentrator. The out-of-plane sensitivity of the sensor can be further improved by optimization of the flux concentrator and the magnetic core.

## Figures and Tables

**Table 1:** Definition of coil pairs for different sensing modes.

Mode	Coil Pairs
X-axis	1–3
Y-axis	2–4
Z-axis	1–2 and 3–4

**Table 2:** Parameters for the sensor.

Parameters	Values
Excitation coil turns	25 × 1
Induction coil turns	15 × 4
Size	29.8 × 31.9 mm^2^
Hight of the flux concentrator	34.9 mm
Angle of the flux concentrator	60

**Table 3:** Sensor performance for different sensing modes.

Mode	Sensitivity [V/T]	Linear Range [μT]
X-axis	257.1	130
Y-axis	468.8	150
Z-axis	258.8	140

## Data Availability

Data are contained within the article.
